# Bilateral Dacryoadenitis As the First Presentation in an Undiagnosed Sarcoidosis Patient

**DOI:** 10.7759/cureus.48287

**Published:** 2023-11-05

**Authors:** Motaz Saifi, Anas Odeh, Saad Abuzahra, Omar Younis, Yousef Shanti

**Affiliations:** 1 Department of Medicine, An-Najah National University, Nablus, PSE; 2 Department of Ophthalmology, An-Najah National University Hospital, Nablus, PSE

**Keywords:** autoimmune, lymphadenopathy, lacrimal gland enlargement, sarcoidosis, dacryoadenitis

## Abstract

Sarcoidosis is an idiopathic multisystem disorder associated with hilar lymphadenopathy and noncaseating granulomas that can affect any organ. Ocular involvement is less common; however, sarcoidosis is a known cause of uveitis, dry eye, and conjunctival nodules. We report a case of a 36-year-old male with an occupational history of dust exposure presenting to the ophthalmology clinic with bilateral painless upper eyelid swelling of one-week duration. The diagnosis of sarcoidosis was suspected based on clinical examination, laboratory analysis, and imaging showing mediastinal lymphadenopathy, further confirmed by pathologic examination showing noncaseating granulomas with the presence of some asteroid and Schaumann bodies. A treatment plan consisting of prednisone, folic acid, and azathioprine was effective for the patient, though azathioprine was eventually changed to methotrexate due to an allergic reaction. The patient is on a maintenance dose of methotrexate and is asymptomatic after a year of careful management and follow-up. This case emphasizes the significance of considering sarcoidosis as a differential diagnosis in patients presenting with bilateral dacryoadenitis.

## Introduction

Sarcoidosis is a complex, multisystem inflammatory disease characterized by the formation of noncaseating granulomas in various organs. Although it primarily affects the lungs and lymph nodes, sarcoidosis can involve multiple organ systems, including the eyes. Ocular involvement can be the presenting symptom in up to 30% of patients, most commonly as uveitis, dry eye, and conjunctival nodules [1].

Dacryoadenitis is the inflammation of either the main or accessory lacrimal glands. It is often caused by infections and typically affects one eye. Infections can arise from the conjunctiva, skin, trauma, or bacteremia. Viruses, especially Epstein-Barr virus, adenovirus, herpes simplex, and herpes zoster, are the primary culprits in viral nonsuppurative dacryoadenitis [2], while bacterial infections, primarily *Staphylococcus aureus*, *Streptococcus pneumoniae*, and gram-negative rods, tend to lead to suppuration. In rare cases, fungal sources like Histoplasma, Blastomyces, or Nocardia may be responsible. In addition to infections, dacryoadenitis can be associated with autoimmune disorders such as thyroid eye disease, granulomatosis with polyangiitis, Sjogren's syndrome, and IgG4-related disease [3]. Neoplastic causes, including lymphoma, adenoid cystic carcinoma, and pleomorphic adenoma, are also possible causes.

Although uncommon, it can manifest as an isolated ocular condition or as part of a systemic disease in cases of sarcoidosis [4]. While unilateral involvement of the lacrimal glands is more prevalent, bilateral involvement can also occur, presenting diagnostic challenges, particularly when sarcoidosis is not initially suspected.

We present a rare initial manifestation of sarcoidosis as bilateral dacryoadenitis in a previously healthy 36-year-old male patient.

## Case presentation

**Figure 1:**
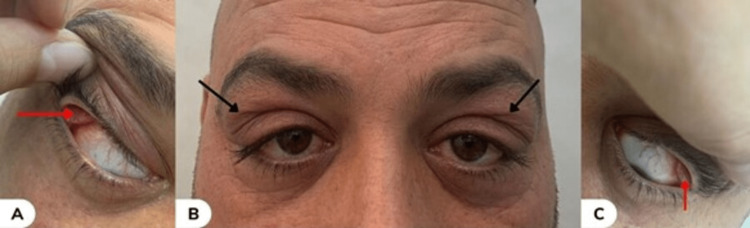
(A) Enlargement and protrusion of the palpebral part of the right lacrimal gland (horizontal red arrow); (B) Front view showing the enlargement in the upper eyelids of the patient (black arrows); (C) Enlargement and protrusion of the palpebral part of the left lacrimal gland (vertical red arrow)

A 36-year-old male presented with a one-week history of bilateral painless upper eyelid swelling (Figure 1). The patient is a 10-pack-year smoker and works as a building contractor, regularly encountering dust in his occupational environment, with no significant medical or family history.

**Figure 2:**
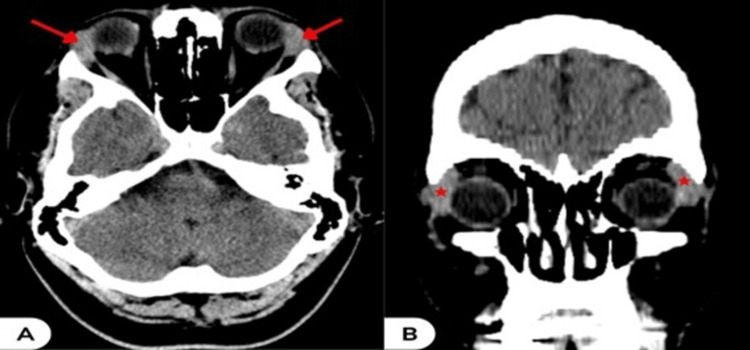
(A) Axial view head CT showing bilateral lacrimal gland enlargement (red arrows); (B) Coronal view (red stars)

**Figure 3:**
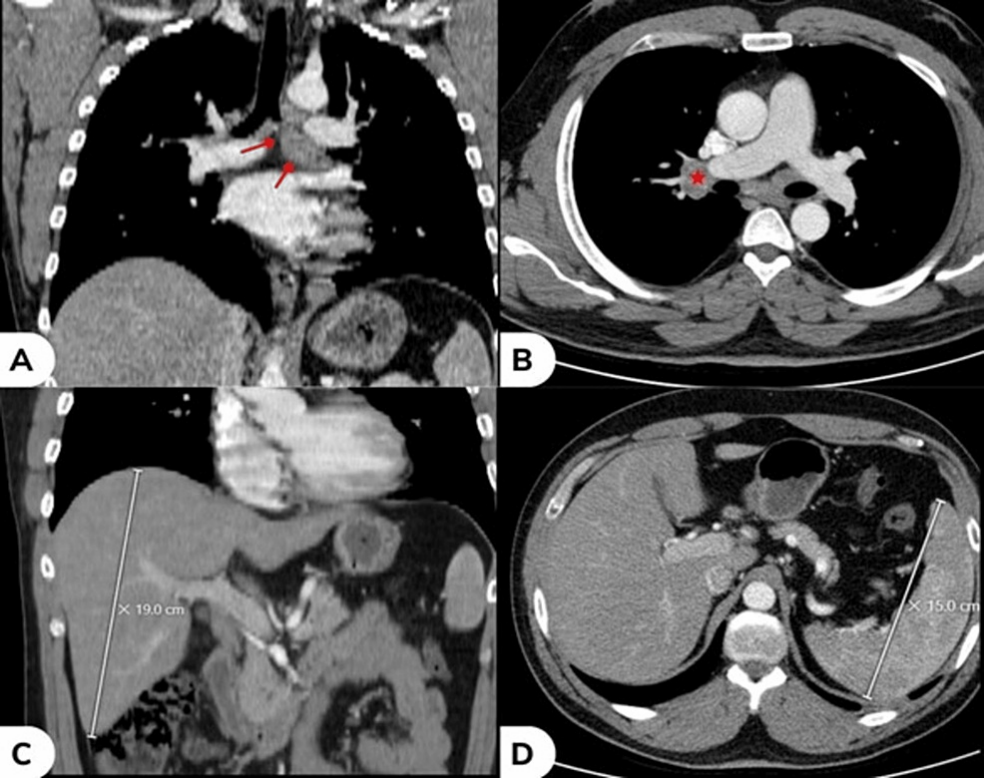
(A) Coronal view CT showing subcarinal lymphadenopathy (red arrows); (B) Axial view CT showing right hilar lymphadenopathy (red star); (C) Coronal view CT showing enlargement of the liver measuring 19.0 cm (white meter); (D) Axial view CT showing enlargement of the spleen measuring 15.0 cm (white meter)

Clinical examination revealed hyperemic eyes with papillary conjunctivitis, mild upper eyelid edema, and palpable, non-tender, solid swelling at the palpebral parts of both lacrimal glands. Ophthalmic examination showed normal visual acuity, visual field, pupillary light reflex, intraocular pressure, and eye movement. Slit-lamp biomicroscopy, fundoscopy examination, and Schirmer's test were also normal. The patient was treated as a case of allergic conjunctivitis for four days without improvement. At the follow-up visit, comprehensive laboratory analysis showed elevated calcium levels of 11.1 mg/dl (normal range: 8.6-10 mg/dl) and the acute phase reactant C-reactive protein (CRP) of 96 mg/dl (normal range: <6 mg/l). However, a complete blood count, erythrocyte sedimentation rate (ESR), liver function tests, kidney function tests, thyroid function tests, parathyroid hormone levels, creatine kinase levels, and urinalysis, as well as hepatitis B surface antigen, all yielded normal or negative results. This was followed by whole-body computed tomography (CT), which revealed the presence of bilateral lacrimal gland enlargement (Figure 2), mediastinal lymphadenopathy (Figures 3A, 3B), mild enlargement in the liver (Figure 3C), and an incidentally enlarged spleen (Figure 3D).

Based on the previous imaging findings, a bronchoscopy and mediastinal lymph node biopsy were performed for pathological investigation due to suspicion of sarcoidosis or lymphoma. The microscopic examination showed non-caseating granuloma with the presence of some asteroid and Schaumann bodies, which are consistent with a diagnosis of sarcoidosis.

The patient was prescribed prednisone, folic acid, and azathioprine, which was soon replaced with methotrexate due to a possible allergic reaction characterized by continuous hot flashes related to azathioprine. Methotrexate was initiated at an initial dosage of 40 mg/day and gradually tapered down to 5 mg/day over a two-month period, during which significant improvement was observed.

During follow-up, the patient reported experiencing blurred vision, and a slit lamp examination revealed the presence of uveitis. As a result, the dosage of methotrexate was increased to 40 mg/day until the symptoms improved, after which it was gradually tapered. After one year of careful management and follow-up, the patient is currently asymptomatic and taking a daily maintenance dose of methotrexate (5 mg/day), in addition to folic acid (5 mg, day after day) and cortisone (5 mg, day after day).

## Discussion

Sarcoidosis is a chronic inflammatory condition marked by the presence of noncaseating granulomas in various organs throughout the body. These granulomas, which consist of aggregates of immune cells such as lymphocytes and macrophages, accumulate within affected tissues [5]. Although sarcoidosis was first described in the late 18th century, the precise cause of this systemic disease has yet to be fully understood [6].

Sarcoidosis is known to affect patients of all ages and ethnicities. However, higher rates of incidence have been reported in patients of African-American descent [7]. In the United States, the incidence of sarcoidosis is reported to be approximately 11 cases per 100,000 individuals among Caucasians. The incidence was found to be higher among African Americans, with around 34 cases per 100,000 individuals [7].

The pathophysiology of sarcoidosis is characterized by the formation of non-caseating granulomas with the presence of macrophages and multinucleated giant cells. Despite the well-studied mechanisms underlying granuloma formation, the exact cause is still poorly understood. It has been observed that certain occupational and environmental factors, such as exposure to beryllium, dust, and other substances that can induce asthma, may be linked to the development of this condition [8,9]. Some studies suggest a potential infectious cause; such studies showed associations between sarcoidosis and various microorganisms, including mycobacteria and propionibacteria [10].

The clinical presentation of sarcoidosis varies significantly from one patient to another, with diverse manifestations and symptom severity. Sarcoidosis is often discovered incidentally in patients with no symptoms or non-specific complaints. In symptomatic patients, the clinical manifestations are usually organ-specific due to the multisystemic nature of this disease. Common symptoms seen in sarcoidosis are a persistent dry cough, fatigue, and shortness of breath [11]. Organ-specific symptoms may include painful red skin nodules, uveitis leading to blurred vision, hoarseness of voice, palpable lymph nodes in various locations, including the armpits and neck, painful joint swelling, hearing loss, seizures, and potential psychiatric disorders as part of neurological involvement [12].

In sarcoidosis diagnosis, it is crucial to consider the appropriate clinical presentation, the presence of nonnecrotizing granulomatous inflammation in one or more tissue samples, and the exclusion of other causes of granulomatous diseases when using diagnostic modalities, such as laboratory testing, imaging methods, and invasive procedures [13]. To assess organ involvement and disease activity, laboratory procedures such as complete blood counts, inflammatory markers like C-reactive protein (CRP), and liver function tests are utilized. Additionally, particular symptoms, like bilateral hilar lymphadenopathy and pulmonary infiltrates, may be detected using imaging modalities such as chest X-rays, computed tomography (CT) scans, and magnetic resonance imaging (MRI) [14].

In treating sarcoidosis, the primary objective is to tackle organ dysfunction, prevent permanent scarring, and enhance the overall quality of life [14]. Corticosteroids are generally the first choice for therapy; however, prolonged use can result in significant complications. In situations where therapy fails or toxic side effects arise from corticosteroid use, second-line therapy like methotrexate or azathioprine can be used. Third-line therapy may include tumor necrosis factor-alpha (TNF-alpha) inhibitors or interleukin-6 (IL-6) antagonists like infliximab or adalimumab [15].

Lacrimal gland inflammation is known as dacryoadenitis. It may manifest isolated or in conjunction with several underlying conditions, sarcoidosis being one of them. Symptoms include pain, swelling, tenderness in the upper outer corner of the eye, and, less commonly, tearing and redness. The disorder can be acute or chronic, with the chronic form being linked to autoimmune diseases like sarcoidosis or Sjogren's syndrome. A thorough medical history, physical examination, and imaging tests are used to identify dacryoadenitis. Depending on the underlying reason, treatment may involve conservative measures in addition to systemic or local anti-inflammatory drugs [16].

In our case report, the patient presented with bilateral dacryoadenitis as the initial presentation of sarcoidosis. While ocular involvement is not uncommon in sarcoidosis, bilateral dacryoadenitis is extremely rare [1]. In 2017, Roca et al. reported a case of unilateral dacryoadenitis in the right eye as the initial presentation of sarcoidosis. In contrast, our case presented with bilateral dacryoadenitis [4]. Further investigations revealed incidental visceral involvement through imaging. This is in contrast to the case reported by Roca et al., which had no extra-ocular involvement. Similar to our case, Raco et al. reported minimal elevation of inflammatory markers [4]. This may suggest that patients having dacryoadenitis as the initial presentation might have limited elevations in the inflammatory markers. This highlights the importance of maintaining a degree of suspicion for sarcoidosis in patients with unexplained dacryoadenitis.

## Conclusions

Sarcoidosis is a systemic granulomatous disease that can affect any organ. It usually starts in the lungs, skin, or lymph nodes. Ocular involvement, such as uveitis, dry eye, and conjunctival nodules, is less common but can be the presenting symptom in some patients. Dacryoadenitis is an inflammation of the lacrimal glands and can have infectious, neoplastic, or autoimmune etiologies. Therefore, when managing dacryoadenitis, it is important to consider sarcoidosis as one of the potential diagnoses, as it can manifest solely as ocular symptoms.
